# Religiosity and Engagement in Early Intervention Programme for First‐Episode Psychosis

**DOI:** 10.1111/eip.70245

**Published:** 2026-07-31

**Authors:** Alona Schneidman, David Roe, Yaara Zisman‐Ilani, Carmit‐Noa Shpigelman

**Affiliations:** ^1^ Department of Community Mental Health University of Haifa Haifa Israel; ^2^ Department of Psychiatric Rehabilitation and Counselling Professions Rutgers University New Brunswick New Jersey USA; ^3^ Department of Social & Behavioral Sciences Barnett College of Public Health, Temple University Philadelphia Pennsylvania USA; ^4^ Department of Psychiatry & Behavioral Science Lewis Katz School of Medicine, Temple University Philadelphia Pennsylvania USA; ^5^ Department of Clinical, Educational & Health Psychology, Division of Psychology & Language Sciences University College London London UK

**Keywords:** early intervention services engagement, first episode psychosis, NAVIGATE, religiosity

## Abstract

**Background:**

While religious beliefs are known to affect help‐seeking pathways, little is known about how they shape engagement in first‐episode psychosis (FEP) care. The purpose of the present study is to explore how religious belief, religious identity and cultural norms shape engagement with FEP care.

**Methods:**

Semi‐structured interviews were conducted with 23 NAVIGATE participants in Israel. The sample comprised 11 Ultra‐Orthodox (UO) and 12 non‐observant (Non‐Obs) Jewish FEP participants. Thematic analysis was used to identify themes related to religious factors in FEP care engagement and the therapeutic alliance.

**Results:**

Two main themes emerged. The first focused on how faith contributed to participation in NAVIGATE by framing FEP care as God's will and viewing therapy as a spiritual experience. The second focused on trust issues, emphasising autonomy and collaborative decision‐making. Across both themes, the therapeutic relationship emerged as a critical independent source of NAVIGATE engagement.

**Conclusions:**

Findings indicate that religiosity shapes day‐to‐day engagement throughout FEP care, extending beyond initial help‐seeking. Implications are discussed with a focus on dialogue, psychoeducation and communication and on combining religious responsiveness with a strong therapeutic alliance.

## 1 Introduction

First Episode Psychosis (FEP) programmes improve clinical and functional outcomes for individuals with FEP and support reintegration into their communities (Bertulies‐Esposito et al. 2025; Farooq et al. 2024; Kane et al. 2016; Rosenblatt et al. 2025; Russell et al. 2025). Despite this demonstrated positive impact, engagement, defined as attendance, utilisation of FEP programmes and adherence to FEP treatment (Roe, Jones, et al. 2021), remains a persistent challenge (Cowan et al. 2020; Ferrari et al. 2024; Mlay et al. 2025; Robson and Greenwood 2022; Schneidman et al. 2025). Research has identified several factors that influence engagement with FEP care, including individual beliefs about the causes of mental conditions (Rosenthal Oren et al. 2021), proactive family intervention strategies and therapeutic alliance and goal alignment between clinicians and service users (Foo et al. 2026). The purpose of the present study is to explore how religious belief, religious identity and cultural norms shape engagement with FEP care.

Religiosity and spirituality shape how people with psychosis understand distress and recovery and their impact on symptom trajectories (Westhead and Georgiades 2025). Spiritual support, such as finding meaning in illness, drawing comfort and strength from faith, maintaining hope and viewing treatment as compatible with one's religious beliefs, has been associated with higher levels of well‐being and treatment adherence (Westhead and Georgiades 2025). In contrast, spiritual struggle and divine punishment appraisals have been linked to suicidality, poorer functioning and difficulties with treatment engagement or adherence (Rosmarin et al. 2013; Serfaty et al. 2020; Pirutinsky and Rosmarin 2022; Westhead and Georgiades 2025). Religious and spiritual beliefs also shape symptom causal explanation**s** as they can influence coping and help‐seeking behaviours (Peteet 2019; Rosenthal Oren et al. 2021; Sawab et al. 2024; Westhead and Georgiades 2025). Religiosity and spirituality need to be approached carefully in psychiatric treatment: for some, religious beliefs may intensify symptoms, whereas for others, engagement with spiritual resources can foster meaning‐making and support ongoing engagement in care (Jones et al. 2021, 2023; Richardson et al. 2024).

Religiosity is often conceptualised as a lived, relational concept that includes personal religious beliefs (e.g., beliefs about God and healing), religious identity (e.g., seeing care as consistent with being a faithful member of one's religious community) and religious‐cultural norms (e.g., norms around family responsibility, authority and decision‐making) that shape family roles, authority structures and expectations about mental condition and recovery (Cucchi and Qoronfleh 2025; Richardson et al. 2024). Accordingly, religiosity is conceptualised as a system of meaning and authority embedded in family and community contexts and particularly salient during early psychosis, when decisions about care and autonomy are actively negotiated.

Recent research on engagement with FEP care has focused on challenges, including delayed pathways into care, stigma and limited cultural responsiveness in FEP programmes (Hussain et al. 2025). Research has also examined facilitators of engagement in FEP programmes, such as more culturally sensitive, family‐oriented and trust‐building approaches (Benuto et al. 2023; Oluwoye et al. 2018; Michaels et al. 2024; Myers et al. 2023) and approaches tailored for immigrants (Anderson et al. 2015; Hernandez et al. 2019). However, less attention has been paid to barriers and facilitators related to the religiosity dimension among FEP programme participants.

Religious groups constitute distinct subcultures, each with its own values, norms and practices that influence how they recognise psychiatric symptoms, seek help and engage with mental health services (Berkovitch and Manor 2023; Pinchas‐Mizrachi et al. 2024). These factors are particularly salient in FEP contexts, a distinct phase of care characterised by developmental transitions, evolving identity and heightened involvement of family and community members (McGorry et al. 2008). The onset of FEP typically occurs during a developmental period when personal, moral and religious beliefs and identities are still forming, thereby influencing perceptions and experiences of illness, recovery and the role of treatment (Rosenthal Oren et al. 2021). In addition, for some individuals, psychotic experiences may involve religious or spiritual content, such as delusions or hallucinations (Cook 2015), posing challenges for clinical interpretation, therapeutic alliance and ongoing engagement with care (Royal College of Psychiatrists 2025; Westhead and Georgiades 2025). For example, in certain religious communities, women may not be treated by male therapists and fundamental FEP principles, such as collaborative approaches and shared decision making (SDM), can conflict with religious beliefs and norms (Zisman‐Ilani, Barnett, et al. 2017; Zisman‐Ilani et al. 2024). Furthermore, mental health may be viewed as a punishment from the divine, which can influence engagement and adherence to treatment (Boateng et al. 2024; Peteet 2019; Rosenthal Oren et al. 2021).

Notably, although religious beliefs are often discussed in the literature as barriers to help‐seeking, they do not preclude engagement altogether. Many religious individuals do enter public mental health services, including early intervention programmes, often through complex, sequential pathways involving family members and religious authorities (Peteet 2019). However, far less is known about what happens after entry into an FEP programme, specifically how religiosity continues to shape engagement, therapeutic alliances and decisions to remain in or disengage from care.

Ultra‐Orthodox (UO) Jews constitute a religious minority. Like other conservative or traditionalist religious groups, the UO community is characterised by strong adherence to religious law, close‐knit family and community life and reliance on religious authority in everyday decision‐making. A central belief shared across these groups is the worldview of divine providence, the understanding that life events, including illness and recovery, are guided by God's will. Within this framework, mental distress may be interpreted as meaningful or spiritually purposeful and healing is often seen as ultimately dependent on faith, even when professional help is sought. Engagement with modern mental health services may therefore involve ambivalence, particularly when treatment emphasises individual autonomy or SDM, which can conflict with authority‐based religious norms (Doron et al. 2024; Zisman‐Ilani et al. 2020). The purpose of the present study is to explore how religious belief, religious identity and cultural norms shape engagement with FEP care. Focusing on UO and non‐observant (Non‐Obs) Jewish participants, we explored firsthand reflections on the role of religiosity in engagement with NAVIGATE, a FEP programme in Israel.

## 2 Method

### 2.1 Setting and Participants

The study was conducted at four NAVIGATE clinics in Israel. NAVIGATE is a team‐based programme for FEP that provides coordinated, recovery‐oriented care through psychotherapy, medication management, family support and vocational services (Roe, Mashiach‐Eizenberg, et al. 2021).

We focused on two groups of participants who self‐identified as UO or Non‐Obs. Inclusion criteria were: (a) age 18 years or older; (b) experienced a FEP; (c) enrolled in a NAVIGATE programme for at least 3 months within the past 3 years; we considered this period the minimum required for participants to develop a meaningful experience of the programme; (d) having graduated from or disengaged from the programme no more than 3 years prior to participation; and (e) ability to provide informed consent and participate in an in‐depth interview.

### 2.2 Recruitment Procedures and Data Collection

Recruitment took place from November 2021 to April 2023. The NAVIGATE clinical staff identified 67 potential participants who met the inclusion criteria and asked whether they were interested in taking part in the study. Of these, 42 (63%) expressed interest and 23 (11 UO and 12 Non‐Obs) agreed to participate. Participants provided written or oral consent prior to the start of the study. For oral consent, the process was recorded. The study protocol was approved by both the Institutional Review Board of Haifa University, Haifa, Israel and the Helsinki ethics committees of NAVIGATE clinics.

Data was collected through semi‐structured interviews. Interviews were conducted via a video‐conferencing platform and lasted approximately 1 h. All interviews were conducted by the first author, a certified psychotherapist and doctoral student trained in qualitative interview. The interviews were designed to elicit participants' experiences in their own words, without imposing predefined categories. The interview guide was developed specifically for this study and informed by published qualitative interview guides and frameworks used in FEP care and social science, including evaluation studies and the DSM‐5 Cultural Formulation Interview. The interview guide followed a flexible, person‐centred and open‐ended structure of key questions and probes. As part of a broader project, the interview covered seven domains: (1) life before NAVIGATE, (2) onset of mental difficulties, (3) pathways to care, (4) help‐seeking actions/behaviours during the untreated psychosis (DUP), (5) personal explanations of the mental health condition, (6) experiences of NAVIGATE and (7) post‐NAVIGATE trajectories. The present manuscript focuses solely on the sixth domain from a cultural perspective. Two pilot interviews were conducted to refine the question flow and relevance. Following the pilot, minor revisions were made to the interview guide. Thus, these two interviews were included in the final analysis.

Data were collected during the COVID‐19 pandemic; therefore, participants were offered a choice between face‐to‐face and video‐based interviews. Although some UO communities restrict digital media use (Shpigelman et al. 2021), the UO participants in this study were already engaged with a public FEP programme and had permitted access to technology for health‐related purposes. Nevertheless, individuals from more insular UO groups who avoid digital platforms entirely may be underrepresented in this sample. All participant interviews were audio‐recorded and transcribed verbatim.

### 2.3 Data Analysis

Inductive thematic analysis was applied following the six phases of Braun and Clarke (2012). The first author served as the primary coder. The process began with repeated readings of the transcripts to achieve deep familiarity with the data, alongside reflexive memo‐writing to capture initial impressions and emerging ideas (Phase 1). Meaningful units of text were then systematically coded in ATLAS.ti, attending to both shared patterns and divergent experiences across participants (Phase 2). Codes were subsequently reviewed, compared and organised into preliminary categories (Phase 3). Through iterative comparison, these categories were refined into broader candidate themes that captured participants' lived experiences of engagement with NAVIGATE (Phase 4). Themes were then reviewed, refined and clearly defined to ensure internal coherence and distinctiveness across the dataset (Phase 5). Throughout the analytical process, attention was given to producing a clear, theoretically informed account of the findings (Phase 6). Responses from UO and Non‐Obs participants were analysed comparatively, as group differences were an explicit analytic focus; therefore, the primary coder was aware of each participant's group affiliation. To enhance rigour, all co‐authors audited the developing themes and contributed to refining the final thematic structure. Areas of ambiguity were discussed collaboratively until consensus was achieved.

## 3 Results

### 3.1 Descriptive Statistics

The total sample (*n* = 23) consisted of seven women (30%) and had an average of 19.2 months in NAVIGATE and 18.8 months since graduation from NAVIGATE (see Table 1).

**TABLE 1:** Sociodemographic and self‐report clinical variables for the UO and Non‐Obs groups.

Variables	UO	%	Non‐Obs	%	*p*
Number of participants (*n*, % of total study population)	11	48%	12	52%	
Age in years (Mean ± SD)	28.2 ± 6.3		26.2 ± 5.2		0.38^a^
Gender (Male)	7	64%	9	75%	0.56^b^
Educational level					
High school	2	18%	7	58%	0.11^b^
Higher education/Postsecondary education	3	25%	5	42%	
Diagnosis					
Schizophrenia	8	73%	5	42%	0.21^b^
Other psychotic disorders	3	27%	7	58%	
DUP					
DUP (in months) (Mean ± SD)	18.5 ± 7.8		12.5 ± 8.3		0.17^c^
NAVIGATE					
Time in NAVIGATE (in months) (Mean ± SD)	18.8 ± 8.9		19.5 ± 8.2		0.82^a^
Completed 2 years in NAVIGATE	8	73%	9	75%	0.91^b^
Disengaged early	3	27%	3	25%	0.91^b^

*Note:* Significance level set at *α* = 0.05.

^a^ Welch's independent‐samples *t*‐test.

^b^ Fisher's exact test.

^c^ Mann–Whitney U test. All tests were two‐tailed.

As indicated in the table, there was a noticeable trend towards a higher proportion of schizophrenia diagnoses and a longer duration of untreated psychosis (DUP) in the UO group compared with the non‐Obs group, although these differences did not reach statistical significance. Educational level also appeared to differ descriptively, with higher rates of high‐school‐only education in the non‐Obs group. Despite these clinical and educational trends, engagement outcomes were similar. Given the small sample size inherent to qualitative designs (Boddy 2016), group differences were primarily examined descriptively; however, exploratory comparisons were conducted using appropriate inferential statistical tests.

### 3.2 Themes

Analysis of the data generated two main themes. First, participants described faith in God as having a dual role: it can both encourage engagement by providing hope and meaning and hinder it when religious guilt or conflicting beliefs are dominant. Second, religious and cultural differences between NAVIGATE participants and providers may act as barriers to trust and engagement.

#### 3.2.1 Theme 1: The Role of Faith as Both Facilitating and Creating Barriers in Treatment Engagement

Most UO (8 of 11, 73%) reported that religious beliefs facilitated their engagement in treatment and that religion provided a source of meaning and resilience that supported engagement. For example, several participants described how religious beliefs helped them adhere, so that ‘even with the doubts’, they felt God wanted them ‘to stay and keep trying’. One participant, UO‐1, perceived his therapist as a messenger of God, which helped him to continue being engaged in treatment, even while he was confronted with doubts about whether to rely on professional help:

I believe God sent me to this program through the people around me. Even the therapist is part of God's plan. That helps me to stay, even when it felt very hard because it created a conflict between the help I was provided in the program and my belief that all is in God's hands.

Another participant, UO‐2, described: ‘I remind myself that God gave me this chance to work with professionals, so I should use it. It doesn't mean I don't trust God, it means I trust that God wants me to get better through this help’. UO‐3 elaborated how, by asking God to guide the therapist's words, he was able to enter each session with calmness and openness, seeing NAVIGATE as aligned with his spiritual practice: ‘For me, going to therapy is not against God. I pray before every session, asking God to guide the therapist's words. That makes me feel calmer and more open’.

At the same time, most (9 of 11, 82%) UO participants reported difficult, religion‐related feelings of spiritual transgression, evoking shame, self‐doubt and even moral failure. They reported feelings of guilt about seeking professional treatment. They were often influenced by family or community religious beliefs that faith alone should have been enough for healing. Several admitted feeling tortured by guilt for engaging in treatment; they were concerned that their belief in God was not strong enough; some even felt a sense of ‘*betraying*’ God. One participant described it as ‘choosing people over prayer’, while another said, ‘I felt ashamed, like I was betraying my faith just by showing up at the clinic’. For some participants (*n* = 4, 36%), this conflict between faith and engagement in treatment was never fully resolved, leaving them with guilt after each session. UO‐4 described this as follows:

Every time I left a session, I asked myself if I had disappointed my family and God by thinking how could a person possibly help me more than God can. It made it difficult to accept and benefit from the programme.

It seems that responses from family and the religious community often intensified their ambivalence. Several participants (7 of 11) reported that parents or religious authorities expressed scepticism about psychological treatment, emphasising faith‐based healing and discouraging continued attendance. UO‐5 recalled feeling torn when her parents insisted that therapy reflected a lack of true faith, making her feel that attending therapy was spiritually wrong. Nevertheless, the supportive relationship with the therapist helped her feel valued and gave her hope, encouraging her to carry on despite the religious conflict:

My parents told me that going to therapy shows a lack of faith. They said, ‘If you really believe, you should trust that God will heal you.’ It made me feel like every session was somehow wrong in God's eyes. Nevertheless, the trust I built with my therapist made me feel better and valued, which gave me hope that I can get through it.

This quote helps illustrate that clients experience connection, rapport, trust and validation from the therapist, regardless of religious beliefs. These relational elements are fundamental to engagement and demonstrate how clients manage tensions among religious and family expectations, professional support and their own agency.

While most participants overcame their own and their families' initial doubts and challenges to benefit from the programme, five participants (22%) ultimately disengaged because their families saw the programme as conflicting with their belief system. In these cases, external pressure compounded internal conflict, making engagement increasingly difficult. As one participant, UO‐6, noted, ongoing pressure from his father weakened his confidence in the programme and eventually led to his withdrawal:

My father kept telling me to stop going to NAVIGATE. He said, ‘You don't need it, because it's not doing more for you than God already does.’ Eventually, I felt I had to listen to him and I left the programme.

Religious authority figures also affected participants' struggles with engagement. For some, guidance from their community leaders reinforced doubts about the legitimacy of psychological treatment, intensifying inner conflict. UO‐7 shared how his religious leader's warning that overreliance on professional treatment might weaken his trust in God added weight to this doubt. This made his engagement with NAVIGATE increasingly difficult and ultimately led to his disengagement from the programme. As he described:

I often wondered, why am I sitting with a therapist when prayer should be enough? My rabbi (religious leader) told me that too much focus on psychology takes me away from relying on God. That made it very hard for me to continue and I left the programme.

In summary, the theme reveals interrelated sub‐conflicts that shape clients' therapeutic engagement: conflict with faith (questioning whether professional help contradicts religious beliefs), conflict with God (experiencing guilt or perceived betrayal when seeking therapy) and conflict with family/religious leaders' authority (navigating pressures to adhere to community norms and guidance from parents or religious leaders). Within these tensions, the eventual reliance on God often functions as a coping mechanism rather than as a direct driver of engagement. Importantly, most participants emphasised that faith did not simply facilitate or hinder engagement, but often did both simultaneously. As one participant explained, ‘Faith kept me here and almost pushed me away at the same time’. Fundamentally, what enables clients to navigate these conflicts is the therapeutic relationship. Indeed, some clients build a meaningful connection with their therapist that provides the foundation for working through religious and family expectations. Understanding these distinct yet interconnected conflicts and the role of the therapeutic bond in mediating them offers a more nuanced picture of how religious clients engage with mental health services.

#### 3.2.2 Theme 2: Therapeutic and Familial Attitudes as Barriers to Engagement

This theme was raised only by UO participants. Non‐Obs did not report similar concerns about religious‐cultural differences or family and community objections to care. They objected not to the treatment itself but to specific aspects of how care was delivered. Encouragement to make decisions independently or ‘together with the therapist’ was sometimes perceived by families as undermining legitimate religious authority rather than supporting recovery. Similarly, autonomy‐focused goal‐setting and private therapeutic spaces conflicted with family‐centred norms in which young adults remain embedded within parental responsibility and communal oversight. In addition, psychoeducation framed primarily through biomedical or psychological explanations was perceived as dismissive when religious interpretations of distress were corrected rather than acknowledged, leading to mistrust, reduced involvement, and in some cases, disengagement.

Most UO participants (*n* = 7, 64%) reported that the shortage of UO professionals in the programme led to trust issues among them, their families and the programme's caregivers. The participants expressed unease about opening up to professionals who did not share or understand their faith and practices. They indicated that being able to speak openly about their religious beliefs, because ‘this is the part of who I am’, would foster trusting relationships and alliances. When asked directly, participants said they would have preferred to be asked about their religious or spiritual beliefs and their role in their lives, rather than have those beliefs assumed to be irrelevant or incompatible with treatment.

##### 3.2.2.1 Theme 2.1 Provider‐Level Barrier: Clinicians Not Fully Acknowledging or Engaging With the Religious Context

For some participants (5 of 11), clinicians tended to prioritise biomedical or psychological explanations, without fully engaging with participants' religious interpretations of their experiences. As a result, these participants felt that important aspects of their worldview were neither adequately recognised nor integrated into care. UO‐5 described this experience as follows:

There were several times I told my therapist that everything that happened to me was the result of God's plan and what God wanted to put me through, but the other side [Therapist] did not take it seriously and said that what I had was nothing to do with God and that he could explain about my condition in a scientific way.

Additionally, families initially attended NAVIGATE sessions to stay close and ensure their loved ones were not exposed to messages that conflicted with their religious values. However, after a few sessions, many found the input less relevant to their lives and questioned whether therapists from such different backgrounds could genuinely offer meaningful help. One participant said that a lack of sensitivity to her religious beliefs made it harder to sustain both her trust in NAVIGATE and her family's willingness to support her involvement. She felt that faith was central to her healing but was not sufficiently acknowledged in treatment. Consequently, her family perceived the programme as exposing her to messages that conflicted with her religious values and ultimately encouraged her to withdraw:

I wish the therapists understood that my faith is a big part of my healing process. I need someone who respects and incorporates my religious beliefs into my treatment […]. We talked about it so many times at home and all my family told me that the best for me is to stop attending NAVIGATE because I am exposed to inappropriate messages that we oppose as we are religious people and also, they did not want to participate in any sessions I had.

Participants hoped that professional care could coexist with or support their faith and that their religious explanations of psychosis and mental health conditions would be acknowledged rather than corrected, as they felt this would foster trust.

##### 3.2.2.2 Theme 2.2 Family‐Or Community‐Level Barrier: Families or Religious Leaders Expressing Concerns About Secular Influences

This sub‐theme captures situations in which families or community figures felt uneasy about engaging with services they viewed as too secular or out of step with their values. These concerns often shaped how families related to care and influenced their willingness to take part in the programme. UO‐9 explained that her family perceived the programme as less aligned with their religious values and traditions. Like other participants and their relatives, her family was uneasy about therapists from different backgrounds and concerned that NAVIGATE would introduce ideas and practices incompatible with their religious beliefs. She said her parents feared the programme was ‘too secular’ and did not adequately respect their traditions. This perception created ongoing tension, as her family believed that continued involvement might not only distance her from her faith but also, through participation in family sessions, expose them to perspectives that clashed with their beliefs. She said:

My family felt that the programme is too secular and does not respect our traditions. They worry that our participation would expose both them and me to ideas that do not fit with our religion. […] For example, my therapists talked about making decisions together with me or even on my own, but in our family, we don't do that, we follow our rabbi's guidance and my parents' decisions. It feels very different from how we were raised and that made my family uncomfortable with the programme.

Participants described feeling caught between NAVIGATE's encouragement to make decisions together or independently and their family's norm of relying on parents and religious leaders' guidance, which shaped their comfort with and trust in the programme. This mismatch also led some to experience NAVIGATE as culturally unfamiliar and, at times, spiritually risky, particularly when sessions encouraged autonomy in goal‐setting and open discussion of sensitive topics.

In summary, differences in religious and cultural backgrounds between participants and providers created barriers to trust and engagement. This reveals a fundamental tension between collaborative therapeutic approaches and authority‐based religious frameworks, underscoring conflicts around autonomy, agency and SDM: essentially, collaboration versus authority. These nuances extend beyond broad notions of ‘cultural difference’ or ‘secular versus religious’ divides and warrant deeper exploration. Furthermore, participants expressed a wish for clinicians to invite discussions of how faith impacts their understanding of distress and recovery and to frame flexible treatment in ways that recognise the role of family and religious leaders, rather than framing independence as the primary goal.

## 4 Discussion

This qualitative study examined how religious belief, religious identity and cultural norms shape engagement with NAVIGATE among UO and non‐Obs Jewish participants. Findings suggest that faith in God played a paradoxical role in the treatment experience. Religious participants described their belief in God as a source of hope, resilience and meaning, motivating them to engage with the programme (Rosmarin et al. 2013; Serfaty et al. 2020). At the same time, many reported guilt and inner conflict, which led some to disengage. Even those who continued reported struggling between their belief that healing through God should be enough and the positive real‐life recovery experience with NAVIGATE, which enabled them to reconcile this tension and continue with the programme.

Religious participants often made sense of treatment through their faith, for example, by describing engagement as ‘God's will’, seeing therapists as guided by God or viewing participation as part of a wider spiritual purpose. This faith‐based meaning‐making was evident even when participants described it in terms of struggle, doubt or moral conflict rather than in positive or explicit terms. Religious participants also emphasised that the therapeutic relationship itself was a powerful reason for staying engaged: feeling trusted, emotionally supported and validated by therapists helped some continue even when religious doubts or family pressure made participation difficult. These experiences may have created a sense of safety that enabled religious participants to better tolerate inner conflict and navigate tensions between faith‐based expectations and professional care, highlighting the therapeutic alliance as a key mechanism supporting engagement. This finding aligns with studies showing that feeling respected, understood and emotionally supported by clinicians is central to engagement, particularly when cultural or family expectations create tension (Eassom et al. 2014; Zisman‐Ilani, Hasson‐Ohayon, et al. 2017). Taken together, these experiences align with growing evidence that positive religious coping predicts better well‐being and adherence, while negative religious coping is associated with poorer outcomes and lower treatment credibility (Bridi et al. 2023; Rohr et al. 2025; Pirutinsky and Rosmarin 2022).

The literature on religiosity in psychosis largely draws on individuals with schizophrenia. Our study extends this by focusing on FEP and shows that faith in God continued to influence participants' daily engagement in NAVIGATE throughout the entire care pathway, whereas previous research has emphasised the role of faith mainly during the journey towards treatment initiation (Boateng et al. 2024; Burns et al. 2011; Oxhandler et al. 2021). In the current study, FEP participants described using faith to make continued attendance feel legitimate and worthwhile, for example, by framing engagement as consistent with God's will, understanding clinicians as instruments through whom God provides help and drawing on prayer and religious practice to cope with distress and stay in care. This emphasis on sustained engagement aligns with evidence from early psychosis services showing that therapeutic alliance, trust and perceived validation are central mechanisms supporting continued participation over time (Browne et al. 2021; Bourke et al. 2021). In this way, faith helped sustain hope and motivation to continue even when doubts or symptoms fluctuated. The same belief system could complicate engagement over time through guilt, uncertainty and pressure from family or religious leaders, sometimes reducing openness in sessions or contributing to disengagement when perceived conflicts intensified. While prior research has documented the dual role of religiosity as both a resource and a source of struggle in psychosis (Westhead and Georgiades 2025), our findings highlight that in FEP care contexts, these dynamics are not limited to symptom interpretation or coping but actively influence ongoing engagement with services.

Participants' accounts also reflect that DUP was longer (though not statistically significant) in the UO group. This may suggest culturally shaped help‐seeking pathways, in which families often first consult religious leaders and private psychiatrists within or close to the community and only later approach public mental health services and FEP care, which may be perceived as outside the community. These sequential pathways may have contributed to delayed entry into NAVIGATE in this sample.

Our findings align with emerging intervention approaches that intentionally integrate religious, spiritual and cultural frameworks into psychosis care. Culturally informed, family‐based interventions and spiritually integrated therapies have shown promise in improving engagement, therapeutic alliance and clinical outcomes among individuals with psychotic‐spectrum conditions. For example, Weisman de Mamani et al. (2023) found that a religiously and spiritually integrated, culturally informed therapy was feasible, acceptable and well tolerated among individuals with serious mental illness. Participants showed improvements in symptoms and functioning, suggesting that integrating spiritual beliefs into treatment can enhance engagement and therapeutic alliance without compromising clinical care. In addition, de Mamani et al. (2020) summarised that culture‐ and family‐based interventions that explicitly incorporate individuals' cultural values, religious beliefs and family structures into treatment for psychotic‐spectrum disorders improve engagement, reduce relapse risk and strengthen family support, particularly among ethnically and religiously diverse populations where standard models may feel culturally misaligned. Such approaches emphasise collaboration with family systems, respect for religious meaning‐making and flexible framing of treatment goals. These elements closely mirror participants' expressed preferences in the present study.

Families often warned participants that exposure to NAVIGATE could risk religious values or introduce ‘inappropriate messages’. In our findings, this scepticism was expressed as an active effort by families or religious leaders to distance participants from FEP programmes, rather than as participants describing the service as alienating. This pattern aligns with the literature, which suggests that when services are perceived as insufficiently attuned to or misaligned with minority values, such perceptions may reduce service use and engagement (Khatib et al. 2023; Reich et al. 2024; Rohr et al. 2025). We found that whether families acted as facilitators or barriers depended on their perception of how well the programme content aligned with their religious beliefs. These insights therefore support calls for culturally tailored family interventions that respect religious traditions while promoting recovery (El‐Khani et al. 2021; Toktas 2024).

In sum, this study used the UO community as a contextual case to illustrate how culturally embedded belief systems and family and community authority structures can shape engagement with care during FEP. The data revealed inherent tensions between collaborative therapeutic approaches and authority‐based religious frameworks, along with several interrelated sub‐conflicts that influence participants' therapeutic engagement: conflict with faith (questioning whether professional help contradicts religious beliefs), conflict with God (experiencing guilt or perceived betrayal when seeking therapy) and conflict with family/religious leader authority (navigating pressures to adhere to community norms and guidance from parents or religious leaders). Fundamentally, what enabled participants to navigate these conflicts was the therapeutic relationship. These relational elements underscore that participants experienced connection, rapport and trust from the treatment team regardless of religious beliefs, revealing that religion is not the only facilitator of engagement, as therapeutic relationships clearly serve a vital role.

This study has limitations. Our examination of religiosity focuses specifically on the UO Jewish community. While some patterns may resonate with other religious minority groups, the findings reflect context‐specific dynamics and should not be assumed to represent religious communities more broadly. Additionally, our UO participants had already successfully entered and engaged with NAVIGATE. Consequently, the findings reflect the experiences of those who ultimately reached public FEP care but may not capture the perspectives of more insular UO individuals who never access such services.

Future research would benefit from longitudinal designs that examine how religious beliefs, interpretations of mental distress and service engagement evolve over time, particularly alongside changes in symptoms, recovery experiences and therapeutic relationships. Such studies should examine how faith shapes ongoing engagement, coping strategies and treatment adherence by clarifying the mechanisms through which faith exerts its sustained influence at different stages of care. In addition, exploring whether and how these faith‐related meanings shift as individuals progress through early intervention could provide important insights into the dynamic mechanisms of sustained engagement and disengagement in FEP care.

## 5 Conclusions and Implications

Considering the profound differences between services guided by evidence and those grounded in religious beliefs, we suggest focusing on helping clients and families incorporate treatment into their belief systems and on clinicians communicating in ways that respect faith‐based frameworks. We recommend that clinicians explicitly facilitate dialogue about how participants understand the relationship between divine will and professional help, collaboratively explore ways to integrate prayer or consultation with religious leaders alongside therapeutic work and view recovery through a broader lens that honours family interdependence and community belonging rather than emphasising individualism alone. Psychoeducation and communication strategies that build on faith‐based interpretations can help create a framework and language that help families and clients to experience care as compatible with religious commitment rather than in competition with it. Furthermore, psychoeducation or interventions that support a ‘meeting in the middle’ approach can help both clients and clinicians navigate conflicts, foster understanding and build hope.

Practical strategies might include matching clinicians with similar backgrounds when possible, incorporating religious frameworks into psychoeducation, providing optional consultations with trusted religious leaders (Doron et al. 2025; Bello et al. 2025) and training clinicians in religious competence, respecting norms such as modesty and confidentiality, which has been shown to improve engagement (McEvoy et al. 2017). From the client and family perspective, FEP programmes can support the agency by helping service users address family or religious community concerns, plan respectful ways to involve trusted figures when appropriate and develop faith‐consistent ways to explain treatment while continuing to pursue recovery goals. This approach aligns with evidence that culturally informed, spiritually integrated interventions improve engagement and outcomes by validating clients' belief systems while maintaining clinical rigour (de Mamani et al. 2020; Weisman de Mamani et al. 2023; Lipskaya‐Velikovsky et al. 2025). Paying attention to how these overlapping tensions play out and to the role of the therapeutic relationship in helping individuals work through them may be useful beyond the specific setting of this study. While the ways in which religion is experienced differ across traditions and communities, the underlying process of navigating faith, family expectations and care is likely relevant in many cultural contexts.

## Funding

This work was supported by The Laszlo N. Tauber Family Foundation.

## Ethics Statement

The relevant Ethics Committees approved the study.

## Consent

All participants provided verbal and written consent to participate.

## Conflicts of Interest

The authors declare no conflicts of interest.

## Data Availability

The data that support the findings of this study are available on request from the corresponding author. The data are not publicly available due to privacy or ethical restrictions.
